# Ophthalmic simulated surgical competency assessment rubric (Sim-OSSCAR) for trabeculectomy

**DOI:** 10.1136/bmjophth-2019-000313

**Published:** 2019-08-18

**Authors:** William H Dean, John Buchan, Fisseha Admassu, Min J Kim, Karl C Golnik, Andrew McNaught, Matthew Burton

**Affiliations:** 1 International Centre for Eye Health, London School of Hygiene and Tropical Medicine, London, UK; 2 Division of Ophthalmology, University of Cape Town, Cape Town, South Africa; 3 Ophthalmology, Gondar College of Medical Sciences, Gondar, Ethiopia; 4 Faculty of Infectious and Tropical Diseases, London School of Hygiene and Tropical Medicine, London, UK; 5 International Council of Ophthalmology, San Francisco, California, USA; 6 Ophthalmology, Gloucestershire Hospitals NHS Foundation Trust, Cheltenham, UK; 7 Moorfields Eye Hospital, London, United Kingdom

**Keywords:** treatment surgery, glaucoma, medical education

## Abstract

**Background/aims:**

To develop, test and determine whether a surgical-competency assessment tool for simulated glaucoma surgery is valid.

**Methods:**

The trabeculectomy ophthalmic simulated surgical competency assessment rubric (Sim-OSSCAR) was assessed for face and content validity with a large international group of expert eye surgeons. Cohorts of novice and competent surgeons were invited to perform anonymised simulation trabeculectomy surgery, which was marked using the Sim-OSSCAR in a masked fashion by a panel of four expert surgeons. Construct validity was assessed using a Wilcoxon rank-sum test. Krippendorff’s alpha was calculated for interobserver reliability.

**Results:**

For the Sim-OSSCAR for trabeculectomy, 58 of 67 surgeons (86.6%) either agreed or strongly agreed that the Sim-OSSCAR is an appropriate way to assess trainees’ surgical skill. Face validity was rated as 4.04 (out of 5.00). Fifty-seven of 71 surgeons (80.3%) either agreed or strongly agreed that the Sim-OSSCAR contents represented the surgical technique of surgical trabeculectomy. Content validity was rated as 4.00. Wilcoxon rank-sum test showed that competent surgeons perform better than novices (p=0.02). Interobserver reliability was rated >0.60 (Krippendorff’s alpha) in 19 of 20 steps of the Sim-OSSCAR.

**Conclusion:**

The Sim-OSSCAR for trabeculectomy, a newly developed and validated assessment tool for simulation glaucoma surgery, has validity and reliability. It has the potential to play a useful role in ophthalmic surgical education.

**Key messages**

**What is already known about this subject?**

- A surgical competency assessment tool has already been developed and validated for live glaucoma surgery.

**What are the new findings?**

- A new surgical competency assessment tool has been developed and validated for use in initial simulation-based surgical training in glaucoma surgery.

**How might these results change the focus of research or clinical practice?**

- Ophthalmology training institutions might focus on the use of simulation-based acquisition of surgical competence before live surgical training.

## Introduction

Glaucoma is the third most common cause of blindness globally after cataract and uncorrected refractive error.^1^ Surgical treatment for glaucoma is considered when medical and laser treatment options are exhausted, inappropriate, or unavailable. In many instances, surgical trabeculectomy is considered as a first-line treatment for moderate to advanced glaucoma. Early surgery can provide lower intraocular pressure (IOP) than medical therapy.^2 3^ A prospective multicentre randomised controlled trial is currently underway to compare the effectiveness of primary medical and primary surgical management for people presenting with advanced glaucoma, the Treatment of Advanced Glaucoma Study.^4^

Surgical education for glaucoma is challenging. Opportunities for trainees are often sparse. In the USA, the mean number of trabeculectomies performed by trainees is four.^5^ Similarly, in sub-Saharan Africa the mean number performed by senior trainees was also four (article under review). This may be due to reluctance of surgeons to perform and patients to accept surgery, driven at least in part by the lack of expectation of improvement in vision and visual field loss. Vision never improves, and often is slightly worse following surgery: a recent meta-analysis showed that visual function (mean deviation and best-corrected visual acuity) drops after surgery, however, the gains from reduced rate of progression balance after 18 months, leaving patients better off.^6^ Moreover, the operated eye may be an only eye, often with good visual acuity. There is recent evidence that visual field loss can improve after surgery reduces the IOP.^7^

A structured curriculum, involving extensive simulation-based training, can assist in introducing trainees to glaucoma surgery.^5^ However, there is a paucity of data on the efficacy of simulation-based surgical education in glaucoma surgery techniques, including trabeculectomy. Therefore, to begin to address this gap, we designed a surgical competency assessment tool for simulated trabeculectomy surgery, based on the International Council of Ophthalmology (ICO) ophthalmology surgical competency assessment rubric (OSCAR) for trabeculectomy.^8^

Surgeons begin their training in a specific technique as ‘novices’, having incomplete knowledge and understanding, approaching a task relatively mechanistically. After time observing, learning and practicing under supervision a novice may progress to being an ‘advanced beginner’, demonstrating situational awareness and a working understanding of what is before them. They tend to see actions as a series of separated steps, and can complete some simpler surgical steps without supervision. A surgeon who is ‘competent’ in a technique has a good working and background understanding, and sees actions in relation to goals, at least partly in context. They may complete work independently to a standard that is acceptable, though it may lack refinement. They are capable of deliberate planning and can formulate surgical routines.^9^ Proficiency and full expertise are considered outside of the scope of this context of simulation-based surgical education in trabeculectomy. Even after an ophthalmology trainee has completed training, there is still a great amount of continued training and experience to be gained in order to become a glaucoma ‘specialist’ and attain a level to be considered and gain recognition as an ‘expert’.^10^

It is towards the stage of ‘competent’ through structured ophthalmic surgical training that this development and use of the ophthalmic simulated surgical competency assessment rubric for trabeculectomy (Sim-OSSCAR) is designed to support. The Sim-OSSCAR is aimed at evaluating the progress made by a trainee towards a basic level of competence, in a simulation environment. Specifically, it addresses the binary question: has the trainee invested sufficient sustained deliberate practice on artificial materials for the trainer to decide it is reasonable to progress to supervised live surgical training?

In medical and surgical education, validity refers to the degree to which an instrument measures what it sets out to measure. Face validity describes whether the simulated tasks resemble those that are performed during a surgical procedure in a real-life situation. Content validity is whether the test resembles a specific skill, not other aspects such as anatomical knowledge. Intergrader reliability is the degree of agreement among different graders, and will provide a measure of consensus.

It is accepted that a unified approach of demonstrating evidence to either support or refute the overall validity of an instrument should be used.^11^ Studies of the assessment of surgical education, training and curricula should have discrete benchmarks as guides: described as face, content, construct, concurrent, discriminative and predictive validity.^12^ There is an even greater need for this in high-stakes assessments such as Board or Surgical College certification examinations. The ICO OSCAR for trabeculectomy has been validated for live surgical performance assessment.^8^ This current study is not aimed at validation of a curriculum nor a high-stakes live surgical assessment.

In this study, we aimed to modify the ICO OSCAR, using it as a starting point for developing a formative and summative assessment tool for simulated ophthalmic surgical training in trabeculectomy surgery.

## Methods

### Trabeculectomy Sim-OSSCAR content revision and development

The ICO OSCAR for trabeculectomy was previously developed by experts at the ICO using a modified Dreyfus scale (novice, beginner, advanced beginner and competent).^8 9^ In this study, we have modified the original ICO OSCAR to develop an assessment and training tool for simulated ophthalmic surgical education in trabeculectomy surgery.

The ICO OSCAR was initially edited to remove content not appropriate for simulation-based surgical training. The OSCAR was further adapted to a modified three-stage Dreyfus scale (novice, advanced beginner, competent). The ‘proficient’ and ‘expert’ steps of the scale were excluded. The draft of the trabeculectomy Sim-OSSCAR was sent electronically to a panel of four international content experts for further amendments to the content and structure of the Sim-OSSCAR. These people were selected for their experience and expertise in performing and teaching trabeculectomy surgery. Responses were collated electronically and synthesised into a final version of the rubric, which was distributed for further review. Amendments suggested by only one of the four experts, and disagreements were discussed until a majority consensus was reached.

The Sim-OSSCAR was designed to be used in conjunction with artificial eyes specifically developed for trabeculectomy by Phillips Studios (Bristol, UK),^13^ which has been in used in training programmes for the past 6 years. It could be used in conjunction with surgical training using animal eyes.

### Face and content validity assessment

The Sim-OSSCAR together with the artificial eye for glaucoma surgery, were presented to delegates at the International Glaucoma Society meeting in Germany. Delegates included expert glaucoma surgeons from around the world, and all were consultant or fellow ophthalmologists with a subspecialty interest in glaucoma. Questions were asked about the trabeculectomy Sim-OSSCAR regarding face and content validity. On a five-point Likert scale, surgeons were asked “Do you think the OSSCAR represents the surgical techniques and skills upon which trainees should be assessed?”. Surgeons were also asked: “Do you think the Sim-OSSCAR (used with the artificial eye) is an appropriate way to assess trainees’ surgical skill?”. Responses on the five-point Likert scale were given a numerical value, entered onto a Microsoft Excel spreadsheet, prior to calculating the mean.

### Interobserver reliability assessment

**Figure 1:**
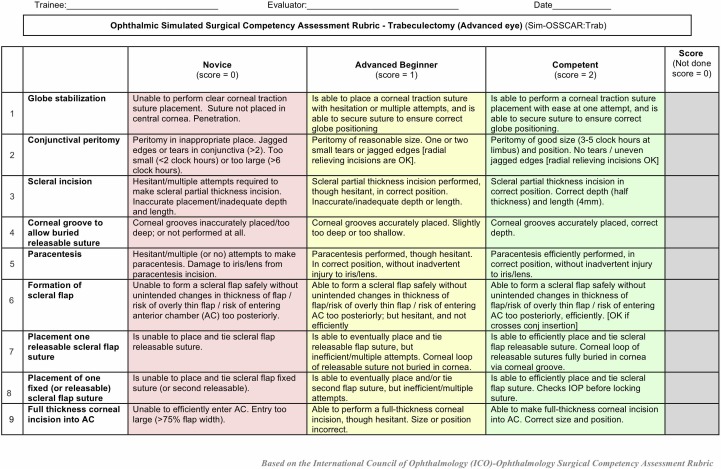
Ophthalmic simulated surgical competency assessment rubric: trabeculectomy (Sim-OSSCAR: Trab). IOP, intraocular pressure.

**Figure 2:**
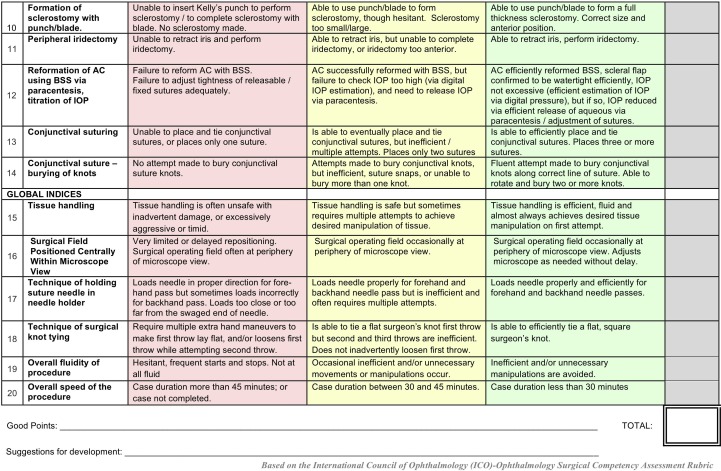
Ophthalmic simulated surgical competency assessment rubric: trabeculectomy (Sim-OSSCAR: Trab). IOP, intraocular pressure; BSS, balanced salt solution .

To assess interobserver Sim-OSSCAR grading reliability we recorded eight simulated trabeculectomy procedures, which were performed by eight separate trainee ophthalmologists. Four were novice trainee surgeons (assisted in less than five trabeculectomies) and four were experienced ophthalmologists (performed more than 100 trabeculectomies). The procedures were performed on the trabeculectomy-specific artificial eye. The simulated surgery was recorded using an Axiocam ErC5reV2 camera mounted to a Stemi 305 desktop microscope (Zeiss, Oberkochen, Germany). The videos were anonymised so that the people doing the scoring were masked to the level of the trainee. The recordings were independently graded using the trabeculectomy Sim-OSSCAR by four ophthalmologists who are highly experienced in trabeculectomy surgery. Expert assessors were masked to the training status of the trainee. Krippendorff’s alpha was calculated for interobserver agreement correlation of the trabeculectomy Sim-OSSCAR ordinal marking scale for each of the 20 sections (figures 1 and 2). Low inter-rater reliability was considered for values of α_k_<0.60.^14 15^ Wilcoxon rank-sum test was performed using the rank sum of the mean scores for novice and competent surgeons. All analysis was performed using Stata V.15.1.

## Results

### Trabeculectomy Sim-OSSCAR content revision and development

The changes arising from the editing of the ICO-OSCAR are shown in table 1. The steps of draping, traction suture, tenons dissection, haemostasis, application of antimetabolite, knowledge of instruments and communication with team were removed. The first stage of ‘globe stabilisation’ included only a clear-corneal traction suture, and not a superior-rectus suture. The expert review group provided feedback on the content of the trabeculectomy Sim-OSSCAR.

**Table 1:** Initial editing of ICO OSCAR for trabeculectomy

Stage of procedure	Action	New Sim-OSSCAR	Comment
Draping	Deleted		
Corneal or superior rectus traction suture	Deleted and edited	Globe stabilisation	
Conjunctival incision and Tenon’s dissection	Deleted and edited	Conjunctival peritomy	Tenon’s dissection deleted
Maintaining haemostasis	Deleted		
Application of antimetabolite	Deleted		
Full thickness incision into anterior chamber (AC)	Added		
Scleral flap suturing/AC reformation	Split into separate sections, edited	Releasable, interrupted sutures	Further AC reformation
Conjunctival closure	Edited	Conjunctival suturing, burying	
Knowledge of instruments	Deleted		
Communication with surgical team	Deleted		
Overall speed and fluidity of procedure	Edited	Fluidity separate Times changes	

ICO, International Council of Ophthalmology; OSCAR, ophthalmology surgical competency assessment rubric; Sim-OSSCAR, ophthalmic simulated surgical competency assessment rubric.

### Face and content validity

Seventy-one surgeons from 22 countries responded to the first question regarding the content of the Sim-OSSCAR, of these 57 (80.3%) either agreed or strongly agreed that the Sim-OSSCAR contents represented the surgical technique of surgical trabeculectomy. The mean content validity was rated as 4.00 (out of 5.00).

Sixty-seven surgeons responded to the second question regarding the face validity of the assessment tool, of 58 (86.6%) either agreed or strongly agreed that the Sim-OSSCAR is an appropriate way to assess trainees’ surgical skill. The mean face validity was 4.04.

### Interobserver reliability

Interobserver reliability was assessed by four expert trabeculectomy surgeons. Eight separate masked video recordings of simulation trabeculectomy were sent to each expert surgeon for scoring using the Sim-OSSCAR. The mean score for ‘novices’ was 4.2 (SD 0.9) and mean for ‘competent’ trabeculectomy surgeons was 33.4 (SD 1.8), out of a maximum score of 40.

To assess the interobserver agreement on the specific items in the Sim-OSSCAR, we calculated Krippendorff’s alpha. A value of 0.60 was deemed acceptable for newly developed rubric.^14 15^ Table 2 illustrates the results for all 20 items in the Sim-OSSCAR, of which 19 exhibited an inter-rater agreement coefficient of α_k_>0.60. Only the positioning of the microscope view had a α_k_<0.60.

**Table 2:** Inter-rater Krippendorff’s alpha correlation for 20 facets of the Sim-OSSCAR

	Item	Krippendorff’s alpha	Per cent agreement
1	Globe stabilisation	0.902	0.934
2	Conjunctival peritomy	0.666	0.792
3	Scleral incision	0.895	0.938
4	Corneal groove(s)	0.782	0.875
5	Paracentesis	0.880	0.934
6	Formation of scleral flap	0.782	0.875
7	Releasable suture	0.755	0.854
8	Fixed/releasable suture	0.635	0.750
9	Corneal incision into AC	0.665	0.771
10	Sclerostomy	0.796	0.875
11	Peripheral iridectomy	0.782	0.875
12	Reformation of AC	1.000	1.000
13	Conjunctival suturing	0.696	0.792
14	Suture burying	0.673	0.792
	**Global indices**		
15	Tissue handling	0.787	0.854
16	Surgical field positioned centrally within microscope view	0.512	0.667
17	Needle holding	0.665	0.771
18	Knot tying	0.639	0.771
19	Overall fluidity of procedure	0.743	0.854
20	Overall speed of procedure	1.000	1.000

AC, anterior chamber; Sim-OSSCAR, ophthalmic simulated surgical competency assessment rubric.

### Construct validity

Construct validity is an assessment of the ‘sharpness’ of a tool: can it discriminate between two distinct groups. For this study these groups are the novice and competent surgeons. Table 3 illustrates the total score for each separate grader for all eight videos.

**Table 3:** Total score correlation

Video	Grader score: n/40					
	A	B	C	D	Mean	SD
1	2	0	1	3	1.50	1.29
2	0	1	0	1	0.50	0.58
3	14	12	14	14	13.50	1.00
4	0	2	0	3	1.25	1.50
5	34	32	32	28	31.5	2.52
6	38	36	36	39	37.25	1.50
7	29	29	32	29	29.75	1.50
8	37	35	36	33	35.25	1.71

Videos 1–4 were performed by novice surgeons, 5–8 by competent surgeons. Scores were out of a possible total of 40. Four expert surgeons (A, B, C and D) graded all eight videos independently.

Novice surgeons were graded with a mean score range of 0.50–13.5 (out of 40), with SD varying between graders’ scores of 0.58–1.5. Competent surgeons were graded with a mean score range of 29.75–37.25 (SD varying from 1.50 to 2.52). A Wilcoxon rank-sum test showed that competent surgeons perform better than novices (p=0.02).

## Discussion

Glaucoma remains a major cause of vision impairment and blindness globally. Four million people have moderate or severe vision impairment, and 2.9 million are blind from glaucoma.^1^ Despite this major burden of disease, trainee eye surgeons perform few glaucoma surgeries during training. There are many challenges in surgical education, with increasing demands for patient throughput, and reducing opportunities for trainees’ hands-on experience.^16^ These challenges are global. If adequate experience cannot be gained through operating, effective adjuncts should be found.

There has been an increase in the use of simulators in ophthalmic surgical training in the past years.^17–19^ This offers an environment in which learners can train until they reach specified levels of competency.^16^ Through simulation-based surgical education, permission to fail can be built into the learning process without risking patient safety. This is especially important in intricate and challenging microsurgical procedures such as trabeculectomy. Furthermore, patients may present with advanced glaucoma, having already lost the vision in one eye. Many glaucoma surgeries are performed on a patient’s only eye.

A trainee should proceed to supervised surgery training on patients in theatre only after having attained a level of competence in the simulated setting. Therefore, a structured training programme needs to include the formal assessment of the performance of simulated surgery, using a validated tool such as the trabeculectomy Sim-OSSCAR. The specific aim of this training and assessment rubric is to help train an eye surgeon who is a novice in trabeculectomy, to a competent level, such that they can commence supervised live surgical training.

The trabeculectomy Sim-OSSCAR has good interobserver reliability. The one step of the rubric to be rated less than 0.6 was ‘surgical field positioned centrally within microscope view’. This is likely due to the limitation of the Zeiss Stemi305 microscope which has a higher zoom when recording, relative to the surgeon’s binocular view. Therefore, the recorded image does not fully reflect the surgeon’s experience.

There are limitations with the use of the Sim-OSSCAR. Its use should be flexible depending on the simulation environment. For artificial eyes, certain amendments or allowances could be made. These may include adding additional text:

- Toothed forceps for peripheral iridectomy (PI) (rather than micro-notched or suture tying forceps).
- Use of larger sutures (8-0) for scleral and conjunctival suturing, and allowances for slipping.
- Larger sclerostomy (than the 0.5 mm or 1 mm in live surgery).
- Flap should be measured from the limbus, and not the conjunctival insertion (which is usually 1–2 mm form the limbus due to a small band of glue which secures the silk mesh used to simulate conjunctiva). Furthermore, the conjunctival sutures would therefore traverse the middle of the scleral flap.
- Conjunctival suture ‘burying’ includes starting the suture from underneath the conjunctiva.

The Sim-OSSCAR should aid initial acquisition of competence for the novice glaucoma surgeon. The goal should be to use it as a formative assessment tool within a simulation-based surgical training programme for trabeculectomy, to take a novice surgeon to the stage of competent. It could then be used as a summative assessment tool to give the green light to proceed to supervised live surgical training. It would be up to individual ophthalmic surgeon trainers or training institutions to benchmark appropriately. A guide could be a mean total score of 80% over three simulated cases, and none of the 20 individual steps scoring a ‘zero’.

We anticipate that this newly developed and validated competency assessment tool will help trainees and trainers in overcoming the challenges of training in glaucoma surgery. Further rigorous validation studies should be conducted for the educational curricula for glaucoma surgical education as a whole.
